# The effect of cannabis edibles on driving and blood THC

**DOI:** 10.1186/s42238-024-00234-y

**Published:** 2024-05-31

**Authors:** S Zhao, B Brands, P Kaduri, C.M Wickens, O.S.M Hasan, S Chen, B Le Foll, P Di Ciano

**Affiliations:** 1 Institute for Mental Health Policy Research, Centre for Addiction and Mental Health, Toronto, Canada; 2 Department of Pharmacology and Toxicology, University of Toronto, Toronto, Canada; 3 Health Canada, Ottawa, ON Canada; 4 Addictions Division, Centre for Addiction and Mental Health, Toronto, Canada; 5 Department of Psychiatry, University of Toronto, Toronto, Canada; 6 Department of Psychiatry and Mental Health, Muhimbili University of Health and Allied Sciences, Dar es Salaam, Tanzania; 7 Campbell Family Mental Health Research Institute, Toronto, Canada; 8 Institute of Health Policy, Management and Evaluation, University of Toronto, Toronto, Canada; 9 Dalla Lana School of Public Health, University of Toronto, Toronto, Canada; 10 Institute of Medical Sciences, University of Toronto, Toronto, Canada; 11 Biostatistics Core, Centre for Addiction and Mental Health, Toronto, Canada; 12 Translational Addiction Research Laboratory, Centre for Addiction and Mental Health, Toronto, Canada; 13 Department of Family and Community Medicine, University of Toronto, Toronto, Canada

**Keywords:** Pharmacology and Toxicology, Public Health, Safety, Addiction Medicine, Cannabis, Cannabis Edibles, Simulated Driving, Blood

## Abstract

**Background:**

Cannabis has been shown to impact driving due to changes produced by delta-9-tetrahydrocannabinol (THC), the psychoactive component of cannabis. Current legal thresholds for blood THC while driving are based predominantly on evidence utilizing smoked cannabis. It is known that levels of THC in blood are lower after eating cannabis as compared to smoking yet the impact of edibles on driving and associated blood THC has never been studied.

**Methods:**

Participants drove a driving simulator before and after ingesting their preferred legally purchased cannabis edible. In a counterbalanced control session, participants did not consume any THC or cannabidiol (CBD). Blood was collected for measurement of THC and metabolites as well as CBD. Subjective experience was also assessed.

**Results:**

Participants consumed edibles with, on average, 7.3 mg of THC, which is less than the maximum amount available in a single retail package in Ontario, providing an ecologically valid test of cannabis edibles. Compared to control, cannabis edibles produced a decrease in mean speed 2 h after consumption but not at 4 and 6 h. Under dual task conditions in which participants completed a secondary task while driving, changes in speed were not significant after the correction for multiple comparison. No changes in standard deviation of lateral position (SDLP; ‘weaving’), maximum speed, standard deviation of speed or reaction time were found at any time point or under either standard or dual task conditions. Mean THC levels were significantly increased, relative to control, after consuming the edible but remained relatively low at approximately 2.8 ng/mL 2 h after consumption. Driving impairment was not correlated with blood THC. Subjective experience was altered for 7 h and participants were less willing/able to drive for up to 6 h, suggesting that the edible was intoxicating.

**Interpretation:**

This is the first study of the impact of cannabis edibles on simulated driving. Edibles were intoxicating as revealed by the results of subjective assessments (VAS), and there was some impact on driving. Detection of driving impairment after the use of cannabis edibles may be difficult.

## Introduction

Use of cannabis edibles is on the rise (Health Canada 2022), with 52% of users reporting use of cannabis in food form in 2022 (Hammond et al. 2022). With increased availability of cannabis through legalization (Health Canada 2022), there has been a rising concern over the impact of cannabis on driving abilities. Epidemiological research into the relationship between cannabis use and driving suggests that there is an increase in the risk of a motor vehicle collision after use of cannabis (Asbridge et al. 2012, Li et al. 2012, Rogeberg and Elvik 2016, Hostiuc et al. 2018, Rogeberg 2019, White and Burns 2021). As a result, some jurisdictions have imposed per se limits on the amount of delta-9-tetrahydrocannabinol (THC; the psychoactive component of cannabis) permitted in blood and/or oral fluid while driving, although these limits vary by jurisdiction (Gjerde and Strand 2023).

Our current understanding of how cannabis impacts driving and the relationship to blood THC is based primarily on the smoked route of administration. However, consuming edible cannabis leads to lower blood THC levels (compared to smoked cannabis) (Newmeyer et al. 2017a, Vandrey et al. 2017, Spindle et al. 2020) due to its lower bioavailability (Chayasirisobhon 2020, Grotenhermen 2003) and first-pass metabolism of THC (Agurell et al. 1986). In addition, blood THC takes longer to reach peak levels and the effects last much longer after edible cannabis compared to the smoked route (Vandrey et al. 2017, Sharma et al. 2012). This is underscored by observations that, despite lower blood THC levels, impairment can be detected following cannabis edibles (Veldstra et al. 2015, Newmeyer et al. 2017b). The investigation of edible cannabis is therefore important, as is an understanding of the pharmacology of cannabis consumed in food form, and ultimately how changes in THC may lead to impaired driving ability (Spindle et al. 2020) and detection of THC in blood.

The purpose of the present study was to investigate the impact of cannabis edibles on driving and blood THC. The results of driving simulator studies suggest that there is a dose-dependent alterations in driving (Ronen et al. 2008, Hartman et al. 2016) after cannabis, suggesting a relationship to blood THC. Studies so far have found increased ‘weaving’ (standard deviation of lateral position; SDLP) (Veldstra et al. 2015, Ronen et al. 2008, Hartman et al. 2016, Bosker et al. 2012, Simmons et al. 2022, Arkell et al. 2019, Arkell et al. 2020, Alvarez et al. 2021, Brands et al. 2021) and changes in both speed (Ronen et al. 2008, Hartman et al. 2016, Simmons et al. 2022, Alvarez et al. 2021, Brands et al. 2021, Di Ciano et al. 2020, Brands et al. 2019, Lenne et al. 2010) and reaction time (Alvarez et al. 2021, Brands et al. 2021, Lenne et al. 2010, Sewell et al. 2009, Hartley et al. 2019) following smoked cannabis. Important questions thus remain as to the impact of cannabis edibles on driving. In Canada, the legal limit of THC per packet of edible is 10 mg. In the present study, participants consumed up to one packet of edibles, providing an ecologically valid test of the impact of legally available cannabis on driving.

## Methods

This study was approved by the Centre for Addiction and Mental Health (CAMH) Research Ethics Board (#042/2021) and the Health Canada Research Ethics Board (2020-043H). The study was conducted at CAMH in Toronto, Canada. Participants were recruited between November 2022 and April 2023, with no follow-up period.

### Participants

Inclusion criteria required participants to be aged 19–79 years, to have held a valid G (can drive any car, van or small truck) or G2 (can drive Class G vehicles but subject to certain conditions such as zero blood alcohol level) Ontario driver’s license for at least 12 months, to self-report use of cannabis edibles at least once in the past 6 months, and to drive at least once per month. Participants were willing to abstain from cannabis for 72 h and from alcohol and other psychoactive/recreational drugs for 12 h, and were not pregnant or breastfeeding. To mitigate potential effects of practice, participants were excluded if they had previously participated in a similar simulator study. Eligibility was confirmed through a telephone conversation or through an online eligibility survey.

### Study design and procedures

This was a within-subjects, counterbalanced study of the effects of edible cannabis on simulated driving and on blood THC levels, conducted at the Centre for Addiction and Mental Health (CAMH) in Toronto from November, 2022 to April, 2023. This study included a total of 3 sessions: one practice session and two test sessions. Test sessions were scheduled at least 72 h apart to ensure abstinence from cannabis and to avoid possible drug carryover effects. Participants were asked to abstain from cannabis for 72 h prior to the test sessions and received the following two conditions in counterbalanced order: 1) cannabis, in which they ingested their preferred cannabis edible; and 2) a control in which participants were given a candy to consume in place of the edible cannabis, either a chocolate or gummy. Attempts were made to match the control candy to the form of the active edible (17 chose a gummy, 3 chose a chocolate, one had a brownie and one a cookie). No attempts were made to blind the participants to treatment. Rather, the control candy was consumed to provide a methodological control and time point at which to time all subsequent treatments (ingestion marked time 0).

### Test sessions (see Fig. 1)

Informed consent was obtained at the beginning of a practice session, during which participants familiarized themselves with the tests and measures and drove the simulator to exclude those who experienced sickness on the simulator. Prior to each test session, breathalyzer (Alert™ J5 model) and saliva sampling (DrugWipe® 5ng/ml cut-off) were used to confirm self-reported abstinence from alcohol and cannabis, respectively. In addition, a 14-panel urine screen was used to confirm that there was no recent use of other recreational/psychoactive drugs. At the start of each test session, participants were asked about symptoms of withdrawal from cannabis, as assessed by the Marijuana Withdrawal Checklist (MWC; scored on a scale from 1 (lowest) to 4 (highest)) (Budney et al. 1999, Budney et al. 2003). A blood sample was also collected at this time to quantify the baseline levels of blood THC, CBD, and metabolites of THC. Participants then completed all baseline pre-drug assessments including simulated driving, cognitive and psychomotor testing, and subjective assessments. Following collection of baseline measurements, participants were instructed to consume the cannabis edible/control candy and were given a 2 h break. A blood sample was collected 120 min after consuming the cannabis edible or control candy. Participants drove the simulator at 120, 240, and 360 min after consumption. Cognitive and subjective assessments followed each drive (cognitive assessments and some subjective assessments to be published in a separate report). The visual analog scales (VAS) were administered at baseline and then again at 30, 60 min and hourly until 7 h after ingestion. The VAS measures were: ‘I like this drug effect’ (DRUG); ‘I feel this effect’ (EFFECT); ‘I feel the good effects’ (GOOD); ‘I feel the bad effects’ (BAD); ‘I feel the rush’ (RUSH). After each drive, participants were asked about their driving ability: How do you think you performed during the driving simulation? Responses were based on a 5-point scale with the following anchor labels: 1) I demonstrated POOR driving skills; to 5) I demonstrated EXCELLENT driving skills. Participants were also asked about their willingness to drive: How willing would you be to drive a real vehicle? Possible responses were based on a 5-point scale with the following anchor labels: 1) Not at all willing to drive a real vehicle; to 5) Very willing to drive a real vehicle. At least 72 h separated each test session. For details of the driving simulator, see our previous studies (Brands et al. 2019, Fares et al. 2022).

### Cannabis

Eligible participants were asked to bring their own legally purchased pre-packaged edible cannabis to use during the study. The amount of THC and CBD consumed was derived from the packaging.

### Driving simulations

Each time driving simulation trials were conducted, participants completed three independent pre-programmed scenarios. The first two scenarios, each lasting about 10 min, were situated on a two-lane rural highway, included a potentially frustrating event (e.g., slow vehicles), and provided the opportunity to speed and race. These scenarios were designed to assess speed and lateral control, which can be precisely measured using the simulator software. Collisions were also recorded by study personnel. To better simulate the cognitive demands of real-world driving conditions, one of these 10-min scenarios was conducted under dual task conditions, whereby the participant was asked to count backwards by 3s from a randomly selected 3-digit number from 700 to 1050.

The third scenario was programmed to measure reaction time in terms of brake pedal latency. This scenario consisted of an endless 4-lane highway where participants were instructed to drive at 100 km/h, while remaining in the second lane to the right. When presented with a stop sign facing them (labelled a ‘true stop sign’), participants were to come to a complete stop as quickly as possible. When presented with a stop sign facing away from them (labelled a ‘false stop sign’), participants were to maintain their speed. During each trial a total of 10 stop signs appeared suddenly at the far right lane, 7 of them were true and 3 of them were false.

Driving Outcomes:

- SDLP: Standard deviation of lateral position is a sensitive measure of the effects of psychoactive drugs on driving. It is measured in centimetres and measures the amount of ‘weaving’, or lane deviation. It gives an indication of the ability of a driver to remain in their lane. It is the measure most consistently affected by cannabis.
- Mean speed (MS): This is the average speed during the drive, measured in km/hr. Participants were asked to maintain a speed of 80 km/hr. When effects of cannabis are seen, it is generally to produce decreases in speed. These decreases are believed to be compensatory, due to the fact that the participant is aware that they are impaired.
- Standard deviation of speed (SDSP): This represents the variability of speed during a drive. Larger numbers mean that the driver was not able to maintain their speed.
- Maximal speed (MAX): This the maximal speed obtained during a drive.
- Brake latency: This is a measure of reaction time. It is the time in milliseconds taken by a participant to hit the brake pedal after a true stop sign appears at the far right lane.
- Number of collisions: This is manually recorded by study personnel during the drives and consists of the number of times a vehicle collides with another car or any other object.

### Data analysis

Primary outcomes were SDLP, MS, SDSP, MAX and reaction time; there were too few collisions to allow for analysis. To account for the correlation of repeated measures on the participants, mixed-effect models using Time (120 min, 240 min, 360 min), Treatment (No Cannabis vs Cannabis), and their interaction as fixed effects, and individual participants as random effects, were adjusted to all outcome measures. The models for the outcome measures also controlled for session order (the sequence of smoking cannabis or no cannabis), baseline blood THC, and the baseline value of the outcome measure. The contrasts of the least square means of the outcome measures between the treatment groups Cannabis-No Cannabis at each time point for driving and blood THC, CBD, and metabolites of THC (11-Nor-9-carboxy-THC (COOH-THC) and 11-hydroxy-THC (THC-11-OH)).

The correlations of SDLP and MS with blood THC in the Cannabis group at 120 min were tested with correlation analysis (Pearson’s Product-Moment correlation). MS was selected for correlational analyses because an overall effect was identified and SDLP was selected because it is the measure most consistently affected by cannabis (Hartman et al. 2016, Alvarez et al. 2021, Di Ciano et al. 2023).

Adjustment for multiple comparisons was applied to the driving outcomes by multiplying the obtained *p* value by 9 (the number of outcomes). For all other analyses, a *p* value of 0.05 was used. To be consistent with the driving measures in the analysis of the VAS, only the time points at 120, 240 and 360 min were analysed (to correspond with the times of the driving assessments).

For self-rated driving willingness and impairment, comparisons were made between the Cannabis and No Cannabis conditions with a Wilcoxon non-parametric test for paired samples, at each time point.

## Results

**Fig. 1:**
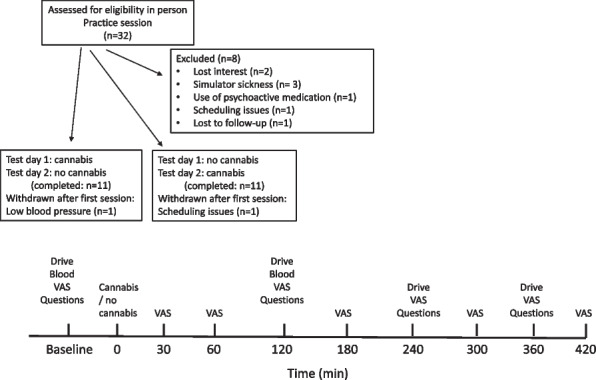
Top figure: CONSORT diagram, illustrating the various test sessions and visits. Bottom figure: Schematic of the test sessions. VAS: Visual Analog Scale; Questions: Driving willingness and impaired driving questions

Participant (*n* = 22) demographics are presented in Tables 1 and 2. A CONSORT diagram is provided in Fig. 1. Participants reported minimal withdrawal symptoms as determined by the MWC (Possible score 1–4; Total score (No Cannabis session: 1.17 (0.24); Cannabis session: 1.16 (0.23)) and the Withdrawal score (No cannabis session: 1.19 (0.25); Cannabis session: 1.17 (0.25)).

**Table 1:** Participant demographics. Mean (SD) and ranges are provided

Male/female	16/6	
Age	47.59 (22.2); 19–74	
**Race/Ethnicity (from a Drop-down menu)**		
White	19	
West Asian or Arab	1	
South Asian	1	
Chinese	1	
**Cannabis Use**		
Years using cannabis	21 (20.7); 1–58	
Primary method to ingest cannabis		
	Joints	6
	Bong	3
	Vapes	3
	Pipes	2
	Edibles	7
	Other	1
Preferred form of edible		
	Gummies	11
	Chocolate	6
	Chocolate and gummies	1
	Brownies and candies	1
	Kief, oil, chocolate	1
	PCB	1
	Powder	1
Frequency of cannabis use		
	More than once a day	4
	Once a day	8
	5–6 times a week	2
	3—4 times a week	3
	Twice a week	1
	Once a week	1
	2–3 times a month	2
	Once every 3–6 months	1
Reason for using cannabis (select the one that applies)		
	Medical	1
	Recreational	19
	Both	2

**Table 2:** Cannabis use by participants in the study. Frequency of use represents the frequency they normally use cannabis. THC (mg) and CBD (mg) represents the cannabis potency consumed in the lab (as derived from the packaging; cannabis with negligible CBD is denoted as 0). Type represents the form of edible consumed in the lab. THC (ng/mL) is the blood THC level at 2 h post consumption of the cannabis edible

Frequency of use	Age	THC (mg)	CBD (mg)	Type	THC (ng/mL)
More than once a day	44	10	0	cookie	5.0
	68	10	0	gummy	4.7
	69	4.5	5.1	gummy	2.2
	73	5	0	gummy	0.6
Once a day	21	4	0	gummy	4.8
	21	10	10	chocolate	4.5
	29	10	10	chocolate	7.8
	35	10	0	gummy	1.9
	71	6	0	gummy	2.3
	72	10	0	brownie	0.6
	74	5	5	gummy	1.3
	74	10	0	chocolate	1.1
5–6 times a week	20	10	0	gummy	6.6
	24	10	0	gummy	3.4
3–4 times a week	24	5	0	gummy	2.4
	45	4	0	gummy	2.3
	67	10	0	gummy	3.9
Twice a week	42	10	0	gummy	3.0
Once a week	23	5	0	gummy	0.7
2–3 times a month	19	5	10	gummy	1.0
	67	2	2	gummy	0.1
Once every 3–6 months	65	5	5	gummy	0.7

In the active condition, participants chose to ingest on average 7.30 (SD: 2.86) mg of THC with 2.14 (SD: 3.65) mg of CBD. Eleven participants ingested cannabis with 10 mg of THC, while 10 chose edibles with 5 mg or less of THC. Fifteen participants chose edibles with negligible CBD, and only two consumed edibles with more CBD than THC (10/5 and 5.1/4.5 mg). Seventeen participants chose gummies, three chose chocolates, one chose a cookie, and one a brownie. Characteristics of the edibles are provided in Table 2. CBD content, deemed as negligible by the manufacturer was reported with various ranges on the packaging; for clarity, cannabis with negligible CBD is denoted as 0.

Significant differences were observed for MS contrasting the least square means at 120 min between the Cannabis and the No Cannabis group under both single task (t(103.82) = -3.04, *p* = 0.003), which was significant after the correction for multiple comparisons (*p* = 0.027), and dual task conditions (t(103.88) = -2.38, *p* = 0.019), which was not significant after the correction for multiple comparisons (*p* = 0.171). No significant effects on other driving measures were found; the number of collisions were too low to allow for analysis. See Table 3.

**Table 3:** Descriptive means (SD) for driving outcomes under single-task conditions (**upper table**) and dual task conditions (**middle table**). Driving outcomes are presented for baseline as well as 120 min, 240 min and 360 min after ingesting cannabis (cannabis) or a control candy (no cannabis) condition. SDLP: standard deviation of lateral position (cm); MS: Mean Speed (km/hr); RT: Reaction time (seconds); SDSP: Standard deviation of speed; Max: maximum speed (km). Descriptive means (SD) of THC, OH-THC, COOH-THC and CBD (**bottom table**) at baseline and 120 min after cannabis or a control condition. *p < 0.05, different from no cannabis for that time point*; + different from no cannabis at that time point (*p* < 0.05), but was not significant after the correction for multiple comparisons

	**Single task**							
	No cannabis				Cannabis			
	Baseline	120	240	360	Baseline	120	240	360
SDLP	30.7 (6.1)	31.2 (6.9)	31.5 (6.4)	31.1 (6.5)	31.6 (7.4)	31.9 (6.9)	32.6 (7.9)	31.7 (7.0)
MS	82.2 (6.1)	82.6 (4.3)	82.2 (3.4)	82.3 (4.6)	81.6 (4.1)	79.8 (4.8)*	82.3 (4.5)	81.9 (4.7)
SDSP	5.1 (2.4)	5.1 (2.5)	5.5 (2.8)	5.5 (2.5)	5.2 (2.5)	5.8 (2.7)	5.0 (2.0)	5.1 (1.9)
Max	95.2 (9.7)	95.3 (9.1)	96.6 (10.2)	95.2 (8.8)	93.7 (7.8)	92.8 (6.1)	95.4 (7.1)	95.0 (7.4)
RT	0.96 (0.11)	0.96 (0.10)	0.97 (0.11)	0.96 (0.10)	0.96 (0.13)	0.96 (0.13)	0.97 (0.10)	0.95 (0.09)
	**Dual task**							
	No cannabis				Cannabis			
	Baseline	120	240	360	Baseline	120	240	360
SDLP	28.3 (5.6)	28.4 (5.0)	29.3 (6.0)	29.3 (5.3)	28.9 (5.7)	30.3 (6.0)	30.4 (6.6)	29.4 (6.8)
MS	83.1 (6.6)	83.7 (6.2)	83.8 (6.0)	83.3 (5.5)	83.0 (5.1)	81.3 (4.4) +	83.3 (5.2)	83.9 (6.0)
SDSP	6.5 (4.0)	6.0 (2.4)	6.7 (3.5)	6.1 (2.5)	5.9 (2.1)	6.4 (2.5)	6.4 (2.6)	6.3 (2.6)
Max	99.2 (9.6)	99.7 (8.8)	100.9 (11.2)	99.8 (8.6)	100.4 (8.6)	98.4 (7.7)	101.4 (8.9)	100.0 (7.9)
	**Blood**							
	No cannabis				Cannabis			
	Baseline	120	240	360	Baseline	120	240	360
THC	0.70 (1.5)	0.89 (1.7)			0.6 (1.0)	2.8 (2.1)*		
THC-COOH	16.7 (34.3)	13.1 (26.4)			8.7 (11.9)	23.6 (22.1)*		
THC-11-OH	.28 (.52)	.28 (.50)			.21 (.28)	2.4 (1.7)*		
CBD	.19 (.21)	.18 (.19)			.17 (.18)	.74 (1.3)*		

Mean blood levels of THC, COOH-THC and THC-11-OH, as well as CBD, increased after ingesting cannabis. Levels were significantly higher in the Cannabis condition compared to the No Cannabis condition at 120 min (THC: t(20.89) = 4.97, *p* < 0.001; COOH-THC: t(21.41) = 5.14, *p* =  < 0.001; THC-11-OH: t(21.17) = 6.59, *p* < 0.001; CBD: t(20.67) = 2.54, *p* = 0.019). See Table 3.

**Fig. 2:**
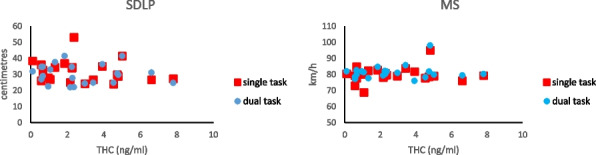
Association between blood THC at 120 min after ingesting cannabis and SDLP (left panel) or MS (right panelunder single-task (red squares) or dual-task (blue circles) conditions

Correlation analysis between THC values at 120 min and driving at 120 min revealed no significant correlations of THC with SDLP (single task: *r* = -0.202, *p* = 0.366; dual task: *r* = -0.096, *p* = 0.671) or with MS (single task: *r* = 0.151, *p* = 0.503; dual task: *r* = 0.139, *p* = 0.536). There were too few cases with CBD to permit analysis of a relationship between driving and blood CBD. See Fig. 2.

**Fig. 3:**
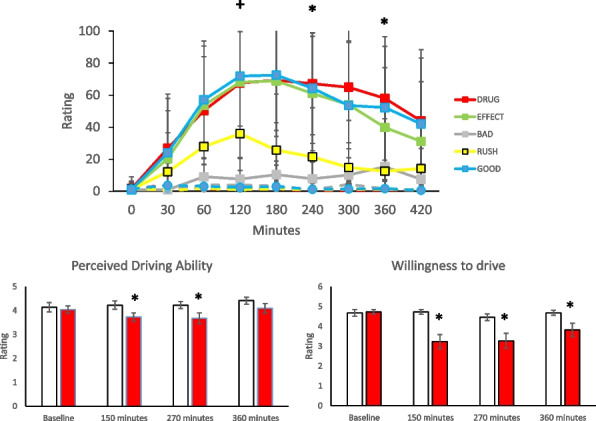
Top panel: Descriptive means (SD) on measures of the Visual Analog Scale. Values are presented for baseline and throughout the session after ingesting cannabis (squares) or after a control candy (circles). + *p* < 0.05 contrasts to the no cannabis condition at that time point for all measures except BAD *p < 0.05 contrasts to the no cannabis condition at that time point for all measures; to be consistent with the driving data only the 120 min, 240 min and 360 min time points were analysed. DRUG: I like this drug effect; EFFECT: I feel this effect; GOOD: I feel the good effects; BAD: I feel the bad effects; RUSH: I feel the rush. Bottom panel: Descriptive mean (SEM) of perceived ability (left panel) and willingness to drive (right panel). Values are presented for the cannabis condition (red bars) or the control condition (open bars). *p < 0.05 contrasts to the No Cannabis condition at that time point

For the VAS, for all measures except BAD, subjective ratings were higher after cannabis at 120 min, as revealed by comparison of least square means between conditions (DRUG: t(104.01) = 10.97, *p* < 0.0001; EFFECT: t(105.41) = 9.94, *p* < 0.0001; GOOD: t(105.1) = 10.59, *p* < 0.0001; RUSH: t(105.37) = 6.36, *p* < 0.0001). All were different at 240 min (RUSH: t(105.37) = 3.78, *p* < 0.001; DRUG: t(104.01) = 11.14, *p* < 0.001; EFFECT: t(105.41) = 8.88, *p* < 0.0001; GOOD: t(105.1) = 9.63, *p* < 0.0001; BAD: t(105.99) = 1.99, *p* = 0.05) and 360 min (RUSH: t(105.37) = 2.08, *p* = 0.04; DRUG: t(104.01) = 9.54, *p* < 0.001; EFFECT: t(105.41) = 5.72, *p* < 0.0001; GOOD: t(105.1) = 7.72, *p* < 0.0001; BAD: t(105.99) = 3.66, *p* < 0.001). See Fig. 3.

When asked how well they had performed during the drive, no differences in participants’ ratings were found between the Cannabis and No Cannabis conditions at baseline. However, ratings were significantly lower in the Cannabis than No Cannabis condition at 150 and 270 min (150 min: Z = -2.06, *p* = 0.040; 270 min: Z = -2.65, *p* = 0.008). When asked to rate their willingness to drive, no differences in participants’ ratings were found between conditions at baseline, but participants were less willing to drive at 150, 270 and 360 min after ingesting cannabis versus control candy (150 min: Z = -3.20, *p* = 0.001; 270 min: Z = -2.93, *p* = 0.003; 360 min: Z = -2.51, *p* = 0.012). See Fig. 3.

## Discussion

This study is the first investigation into the effects of edible cannabis on simulated driving. Subjective effects and blood THC, as well as THC metabolites and CBD were also measured. Consumption of an edible decreased mean speed at 2 h, but not at 4 and 6 h, after ingestion. Relative to the control condition, subjective effects were observed up to 6 h after consumption of the edible and participants reported being less able or willing to drive up to 6 h after consumption. Blood THC and metabolites as well as CBD were increased, relative to control, at 2 h after ingestion of the edible, but blood THC was relatively low at approximately 2.8 ng/mL. Participants chose to ingest on average 7.3 mg of THC, which is less than the amount legally available in a single packet of edibles in Canada (10 mg). The results of this study represent a low dose of THC (Newmeyer et al. 2017a, Vandrey et al. 2017, Spindle et al. 2020, Newmeyer et al. 2017c) and an ecologically valid amount of legally purchased edibles.

The decrease in MS is consistent with our past findings (Brands et al. 2019, Fares et al. 2022) and with the observations of others (Ronen et al. 2008, Hartman et al. 2016, Alvarez et al. 2021, Lenne et al. 2010). It has been suggested that decreased speed after cannabis is a compensatory change in driving (Ward 1999), in response to a participant’s awareness that they are impaired. We have previously found significant increases in SDLP after cannabis of about 2 cm (Fares et al. 2022), but in the present study SDLP was not significantly increased. Other measures, such as reaction time, have also been found to be impacted by cannabis (Alvarez et al. 2021), but were not changed in the present investigation. The lack of effect on these measures may reflect the fact that participants consumed a low dose of an edible. Indeed, driving simulators may not be sensitive enough to measure small changes in performance, as would be observed following low doses of cannabis. Alternatively, the sample size in the present study was relatively small (Brands et al. 2019, Marcotte et al. 2022), but not unprecedented (Bosker et al. 2012), and a lack of effect on other measures may reflect a lack of statistical power. Finally, this is the first study of edible cannabis on a driving simulator and it is possible that edibles have a different impact on driving than that following the smoked or vaped route, upon which current knowledge rests (Simmons et al. 2022, Alvarez et al. 2021, Brands et al. 2021). Future converging evidence from emerging investigations will help to determine the impact of cannabis edibles on driving.

Consistent with our hypothesis, blood THC was significantly increased after consuming the cannabis edible. Mean increases in blood THC were lower than those reported for smoked cannabis (Brands et al. 2019, Fares et al. 2022). Analysis of the relationship of blood THC to SDLP or MS revealed no correlation with blood THC, which fits with emerging evidence from studies of smoked cannabis that there is no linear relationship between blood THC and driving impairment (Di Ciano et al. 2023, Marcotte et al. 2022). It may be possible that, for the smoked route, there is a threshold above which driving is impacted (Di Ciano et al. 2023). However, the present study suggests that blood THC may not be as useful for detection of impaired driving after edibles as it may be for the smoked route (Di Ciano et al. 2023).

Participants in the present study were, for the most part, frequent users of cannabis for recreational purposes. Thus, it is possible that the ‘subtle’ effects observed on driving reflect tolerance to the effects of cannabis. In this regard, evidence for tolerance to the effects of cannabis on simulated driving is mixed. In one study, driving impairments were worse in regular cannabis users compared to non-regular users after smoking cannabis (Downey et al. 2013). In another study, ‘weaving’ was more evident in occasional users, as compared to regular users after oral synthetic cannabis (dronabinol) (Bosker et al. 2012). In a more recent study, occasional users demonstrated more lane departures while distracted, with few differences from habitual users in any other measures while not distracted (Miller et al. 2022). Most previous studies used the smoked route of administration; the edibles route may produce different tolerance, being absorbed through the stomach. In any event, it is interesting to note that, in the present study, there did not appear to be any effects of tolerance on subjective experience. Thus, if tolerance is a consideration in the present study, it may have a different impact on the various outcome measures. Future studies will need to unpack the impact of tolerance on driving after use of edibles.

### Limitations

One limitation of this study is that it does not have any data on body weight and height. Thus, it is not possible to determine whether body mass index (BMI) influenced the impact of the consumed dose on outcomes. Since the edible was taken orally, absorption may have been influenced by BMI. Future large-scale studies will need to determine the relative contribution of BMI to the impact of edibles. Second, the participants in this study were largely white males and thus the results may not be generalizable to the broader public. It is known that it is easier to recruit males in clinical research studies (Mauvais-Jarvis et al. 2021) and white people are generally over-represented in samples. Future studies will need to take this into consideration to apply targeted recruitment strategies to enroll a more representative sample of participants. Third, blinding was not possible in the present study, as participants were invited to consume their preferred edible in the lab. This may have influenced the results, but it should be noted that experienced users of cannabis would likely be able to detect the presence of cannabis in edibles, even with blinding. A fourth limitation is the large age range in this study. It is known that old age has been associated with declines in driving ability (Ball et al. 1993, Owsley et al. 1991, Stutts et al. 1998, Doroudgar et al. 2017, Daigneault et al. 2002, Raedt and Ponjaert-Kristoffersen 2000). Future studies will need to control for age and determine any age-related impacts on the effects of cannabis on driving. This is especially important given that participants over the age of 50 years have been overlooked in studies of the effects of cannabis on driving and related outcomes.

## Conclusion

The present study was the first investigation of the effects of cannabis edibles on simulated driving. Participants were able to choose their own edible and were able to use cannabis to their usual level of intoxication. The present study found edibles produced changes in simulated driving while blood THC levels, although elevated by cannabis, remained relatively low. Legal thresholds of blood THC at the roadside are largely based on research utilizing the smoked route of administration and the present study suggests that the edibles route may be different in important ways.

## Data Availability

The datasets used and/or analyzed during the current study are available from the corresponding author upon reasonable request.

## Abbreviations

CBD: Cannabidiol
COOH-THC: 11-Nor-9-carboxy-THC
MAX: Maximum speed
MS: Mean speed
MWC: Marijuana Withdrawal Checklist
SDLP: Standard deviation of lateral position
SDSP: Standard deviation of speed
THC: delta-9-tetrahydrocannabinol
VAS: Visual analog scale
THC-11-OH: 11-Hydroxy-THC

## References

[CR1] Agurell S, Halldin M, Lindgren JE, Ohlsson A, Widman M, Gillespie H (1986). Pharmacokinetics and metabolism of delta 1-tetrahydrocannabinol and other cannabinoids with emphasis on man. Pharmacol Rev.

[CR2] Alvarez L, Colonna R, Kim S, Chen C, Chippure K, Grewal J (2021). Young and under the influence: A systematic literature review of the impact of cannabis on the driving performance of youth. Accid Anal Prev.

[CR3] Arkell TR, Lintzeris N, Kevin RC, Ramaekers JG, Vandrey R, Irwin C, et al. Cannabidiol (CBD) content in vaporized cannabis does not prevent tetrahydrocannabinol (THC)-induced impairment of driving and cognition. Psychopharmacol. 2019;236(9):2713–24. 10.1007/s00213-019-05246-8 PMC6695367 31044290

[CR4] Arkell TR, Vinckenbosch F, Kevin RC, Theunissen EL, McGregor IS, Ramaekers JG (2020). Effect of Cannabidiol and Delta9-Tetrahydrocannabinol on Driving Performance: A Randomized Clinical Trial. JAMA.

[CR5] Asbridge M, Hayden JA, Cartwright JL (2012). Acute cannabis consumption and motor vehicle collision risk: systematic review of observational studies and meta-analysis. BMJ.

[CR6] Ball K, Owsley C, Sloane ME, Roenker DL, Bruni JR (1993). Visual attention problems as a predictor of vehicle crashes in older drivers. Invest Ophthalmol vis Sci.

[CR7] Bosker WM, Kuypers KP, Theunissen EL, Surinx A, Blankespoor RJ, Skopp G (2012). Medicinal Delta(9) -tetrahydrocannabinol (dronabinol) impairs on-the-road driving performance of occasional and heavy cannabis users but is not detected in Standard Field Sobriety Tests. Addiction.

[CR8] Brands B, Mann RE, Wickens CM, Sproule B, Stoduto G, Sayer GS (2019). Acute and residual effects of smoked cannabis: Impact on driving speed and lateral control, heart rate, and self-reported drug effects. Drug Alcohol Depend.

[CR9] Brands B, Di Ciano P, Mann RE (2021). Cannabis, Impaired Driving, and Road Safety: An Overview of Key Questions and Issues. Front Psych.

[CR10] Budney AJ, Novy PL, Hughes JR (1999). Marijuana withdrawal among adults seeking treatment for marijuana dependence. Addiction.

[CR11] Budney AJ, Moore BA, Vandrey RG, Hughes JR (2003). The time course and significance of cannabis withdrawal. J Abnorm Psychol.

[CR12] Chayasirisobhon S (2020). Mechanisms of Action and Pharmacokinetics of Cannabis. Perm J.

[CR13] Daigneault G, Joly P, Frigon J-Y (2002). Executive Functions in the Evaluation of Accident Risk of Older Drivers. J Clin Exp Neuropsychol.

[CR14] De Raedt R, Ponjaert-Kristoffersen I (2000). The Relationship Between Cognitive/Neuropsychological Factors and Car Driving Performance in Older Adults. J Am Geriatr Soc.

[CR15] Di Ciano P, Brands B, Fares A, Wright M, Stoduto G, Byrne P, et al. The Utility of THC Cutoff Levels in Blood and Saliva for Detection of Impaired Driving. Cannabis Cannabinoid Res. 2023;8(3):408–13. 10.1089/can.2022.0187 36730769

[CR16] Di Ciano P, Matamoros A, Matheson J, Fares A, Hamilton HA, Wickens CM, et al. Effects of therapeutic cannabis on simulated driving: A pilot study. J Concurrent Disord. 2020;2(1).

[CR17] Doroudgar S, Chuang HM, Perry PJ, Thomas K, Bohnert K, Canedo J (2017). Driving performance comparing older versus younger drivers. Traffic Inj Prev.

[CR18] Downey LA, King R, Papafotiou K, Swann P, Ogden E, Boorman M (2013). The effects of cannabis and alcohol on simulated driving: Influences of dose and experience. Accid Anal Prev.

[CR19] Fares A, Wickens CM, Mann RE, Di Ciano P, Wright M, Matheson J, et al. Combined effect of alcohol and cannabis on simulated driving. Psychopharmacol. 2022;239(5):1263–77. 10.1007/s00213-021-05773-3 33544195

[CR20] Gjerde H, Strand MC. Legal limits for driving under the influence of illicit drugs: Large variations between jurisdictions. Forensic Sci Int Rep. 2023;8.

[CR21] Grotenhermen F (2003). Pharmacokinetics and Pharmacodynamics of Cannabinoids. Clin Pharmacokinet.

[CR22] Hammond D, Corsetti D, Fataar F, Iraniparast M, Danh Hong D, Burkhalter R. International Cannabis Policy Study- Canada 2022 Summary. 2023.

[CR23] Hartley S, Simon N, Larabi A, Vaugier I, Barbot F, Quera-Salva MA (2019). Effect of Smoked Cannabis on Vigilance and Accident Risk Using Simulated Driving in Occasional and Chronic Users and the Pharmacokinetic-Pharmacodynamic Relationship. Clin Chem.

[CR24] Hartman RL, Brown TL, Milavetz G, Spurgin A, Pierce RS, Gorelick DA (2016). Cannabis effects on driving longitudinal control with and without alcohol. J Appl Toxicol.

[CR25] Health Canada. Canadian Cannabi Survey. 2022. https://www.canada.ca/en/health-canada/services/drugs-medication/cannabis/research-data/canadian-cannabis-survey-2022-summary.html.

[CR26] Hostiuc S, Moldoveanu A, Negoi I, Drima E (2018). The Association of Unfavorable Traffic Events and Cannabis Usage: A Meta-Analysis. Front Pharmacol.

[CR27] Lenne MG, Dietze PM, Triggs TJ, Walmsley S, Murphy B, Redman JR (2010). The effects of cannabis and alcohol on simulated arterial driving: Influences of driving experience and task demand. Accid Anal Prev.

[CR28] Li MC, Brady JE, DiMaggio CJ, Lusardi AR, Tzong KY, Li G (2012). Marijuana use and motor vehicle crashes. Epidemiol Rev.

[CR29] Marcotte TD, Umlauf A, Grelotti DJ, Sones EG, Sobolesky PM, Smith BE (2022). Driving Performance and Cannabis Users' Perception of Safety: A Randomized Clinical Trial. JAMA Psychiat.

[CR30] Mauvais-Jarvis F, Berthold HK, Campesi I, Carrero JJ, Dakal S, Franconi F (2021). Sex- and Gender-Based Pharmacological Response to Drugs. Pharmacol Rev.

[CR31] Miller R, Brown T, Wrobel J, Kosnett MJ, Brooks-Russell A (2022). Influence of cannabis use history on the impact of acute cannabis smoking on simulated driving performance during a distraction task. Traffic Inj Prev.

[CR32] Newmeyer MN, Swortwood MJ, Abulseoud OA, Huestis MA (2017). Subjective and physiological effects, and expired carbon monoxide concentrations in frequent and occasional cannabis smokers following smoked, vaporized, and oral cannabis administration. Drug Alcohol Depend.

[CR33] Newmeyer MN, Swortwood MJ, Taylor ME, Abulseoud OA, Woodward TH, Huestis MA (2017). Evaluation of divided attention psychophysical task performance and effects on pupil sizes following smoked, vaporized and oral cannabis administration. J Appl Toxicol.

[CR34] Newmeyer MN, Swortwood MJ, Andersson M, Abulseoud OA, Scheidweiler KB, Huestis MA (2017). Cannabis Edibles: Blood and Oral Fluid Cannabinoid Pharmacokinetics and Evaluation of Oral Fluid Screening Devices for Predicting Delta(9)-Tetrahydrocannabinol in Blood and Oral Fluid following Cannabis Brownie Administration. Clin Chem.

[CR35] Owsley C, Ball K, Sloane ME, Roenker DL, Bruni JR (1991). Visual/cognitive correlates of vehicle accidents in older drivers. Psychol Aging.

[CR36] Rogeberg O (2019). A meta-analysis of the crash risk of cannabis-positive drivers in culpability studies-Avoiding interpretational bias. Accid Anal Prev.

[CR37] Rogeberg O, Elvik R (2016). The effects of cannabis intoxication on motor vehicle collision revisited and revised. Addiction.

[CR38] Ronen A, Gershon P, Drobiner H, Rabinovich A, Bar-Hamburger R, Mechoulam R (2008). Effects of THC on driving performance, physiological state and subjective feelings relative to alcohol. Accid Anal Prev.

[CR39] Sharma P, Murthy P, Bharath M (2012). Chemistry, Metabolism, and Toxicology of Cannabis: Clinical Implications. Iran J Psychiatry.

[CR40] Sewell RA, Poling J, Sofuoglu M (2009). The effect of cannabis compared with alcohol on driving. Am J Addict.

[CR41] Simmons SM, Caird JK, Sterzer F, Asbridge M (2022). The effects of cannabis and alcohol on driving performance and driver behaviour: a systematic review and meta-analysis. Addiction.

[CR42] Spindle TR, Cone EJ, Herrmann ES, Mitchell JM, Flegel R, LoDico C (2020). Pharmacokinetics of Cannabis Brownies: A Controlled Examination of Delta9-Tetrahydrocannabinol and Metabolites in Blood and Oral Fluid of Healthy Adult Males and Females. J Anal Toxicol.

[CR43] Stutts JC, Stewart JR, Martell C (1998). Cognitive test performance and crash risk in an older driver population. Accid Anal Prev.

[CR44] Vandrey R, Herrmann ES, Mitchell JM, Bigelow GE, Flegel R, LoDico C (2017). Pharmacokinetic Profile of Oral Cannabis in Humans: Blood and Oral Fluid Disposition and Relation to Pharmacodynamic Outcomes. J Anal Toxicol.

[CR45] Veldstra JL, Bosker WM, de Waard D, Ramaekers JG, Brookhuis KA. Comparing treatment effects of oral THC on simulated and on-the-road driving performance: testing the validity of driving simulator drug research. Psychopharmacol. 2015;232(16):2911–9. 10.1007/s00213-015-3927-9 PMC4513227 25957748

[CR46] Ward NJ DL. Cannabis and driving: a literature review and commentary. DETR; 1999. Report No.: 1468–9138.

[CR47] White MA, Burns NR. The risk of being culpable for or involved in a road crash after using cannabis: A systematic review and meta-analyses. Drug Sci Policy Law. 2021;7.

